# Video-EEG polygraphy in the clinical characterization of hyperkinetic movement disorders: a tertiary referral cohort

**DOI:** 10.3389/fneur.2026.1801440

**Published:** 2026-04-24

**Authors:** Davide Caputo, Brando Rasori, Roberta Solazzi, Laura Canafoglia, Elisa Visani, Davide Rossi Sebastiano, Federica Zibordi, Federica Graziola, Gaetano Cantalupo, Tiziana Granata, Giovanna Zorzi

**Affiliations:** 1 Pediatric Neuroscience Department, Fondazione IRCCS Istituto Neurologico “C. Besta” (full member of the European Reference Network EpiCARE), Milan, Italy; 2 Innovation Biomedicine section, Department of Engineering for Innovation Medicine, University of Verona, Verona, Italy; 3 Department of Diagnostic and Technology, Fondazione IRCCS Istituto Neurologico “C. Besta”, Milan, Italy; 4 Child Neuropsychiatry Unit, University Hospital of Verona (full member of the European Reference Network EpiCARE), Verona, Italy

**Keywords:** electromyographic patterns, neurophysiological phenotyping, pediatric hyperkinetic movement disorders, somatosensory evoked potentials (SEPs), video-EEG–EMG polymyography (P-VEEG)

## Abstract

**Background:**

Hyperkinetic movement disorders (MDs) in children, including tremor, myoclonus, dystonia, chorea, and tics, often present with overlapping or evolving clinical features, making classification based on observation alone challenging. Neurophysiological techniques such as video-EEG polymyography (P-VEEG) and somatosensory evoked potentials (SEPs) may provide objective markers to improve diagnostic accuracy, yet their application in pediatric populations remains limited.

**Objective:**

To describe the polygraphic characteristics of hyperkinetic MDs in a large pediatric cohort and to assess whether specific EMG patterns can support movement disorder classification beyond clinical inspection.

**Methods:**

Seventy-three patients with pediatric-onset hyperkinetic MDs underwent standardized P-VEEG recordings and SEPs at Besta Neurological Institute between January and October 2024. EMG activity was recorded from antagonist muscle pairs during rest, posture maintenance, and specific tasks according to age. Polygraphic findings were compared with clinical diagnoses. Associations between EMG patterns and clinical variables were analyzed using Chi-square tests and regression analyses.

**Results:**

Mean age at MD onset was 9.4 ± 6.1 years and mean age at recording was 13.5 ± 5.1 years. Polygraphic analysis identified four distinct EMG patterns: (i) regular rhythmic oscillatory pattern (RRO), consistent with tremor, in 40 patients (56%); (ii) repetitive myoclonic pattern (RM) in 11 (15%); (iii) complex mixed repetitive pattern (CR) in 9 (12%); and (iv) complex non-rhythmic pattern (CNR), encompassing chorea, dystonia, tics and other dyskinesias, in 11 (15%). Significant differences among repetitive patterns were observed in rhythmicity (*p* < 0.001), burst duration (*p* < 0.001), agonist–antagonist synchrony (*p* < 0.001), and SEP hyperexcitability (*p* < 0.03). RM was significantly associated with enhanced amplitude SEPs (*p* = 0.023), supporting a cortical origin of myoclonus in most cases. A significant association between clinical diagnosis and EMG pattern was found (*p* = 0.003), yet discrepancies between clinical and polygraphic classification was found in 22 cases (30%), particularly among tremor and myoclonus presentations. RM and CR were prevalent in older patients compared with CNR.

**Conclusion:**

P-VEEG proved to be an easily applicable tool providing valuable objective markers for the characterization of pediatric hyperkinetic MDs, revealing distinct EMG patterns that complement clinical evaluation and improve diagnostic precision. Systematic integration of neurophysiological assessment may enhance classification, diagnostic accuracy and help guide management in childhood movement disorders.

## Introduction

Hyperkinetic movement disorders (MDs) in children—including tremor, myoclonus, dystonia, chorea, and tics—often present with distinct clinical features (1), but observation alone does not always allow a reliable categorization of all patients. These disorders can coexist, change over time, and occur alongside other neurological signs (2), highlighting the need for objective, quantifiable tools to assess them and to monitor both disease progression and treatment response. Neurophysiological techniques to study hyperkinetic MDs include surface poly-electromyography (EMG), long-latency EMG responses to nerve stimulation (LLR), electroencephalography (EEG), EEG–EMG polygraphy with back-averaging, cortico-muscular coherence analysis, and somatosensory evoked potentials (SEP) (3–5). These methods support differential diagnosis between myoclonus and other “jerky” movements -sometimes allowing identification of the anatomical generator- and may also help to elucidate the underlying etiologies (6–13, 31). These neurophysiological assessments, while routinely applied in adult populations, especially for characterizing complex movement disorders, are rarely performed in children and their potential to define and manage pediatric hyperkinetic MDs is still scarce (14). In addition, there is limited evidence that the terminology and reference values established for adults are applicable to the pediatric population (15, 16). The purpose of our study is to describe the polygraphic characteristics of hyperkinetic MDs in a large pediatric cohort and to assess whether identification of specific polygraphic patterns can be useful for MD classification. Finally, we aim to compare the polygraphic findings with the clinical diagnosis, to highlight the added value of neurophysiological techniques in the clinical assessment.

## Materials and methods

Seventy-three patients with MDs, with onset in pediatric age, underwent neurophysiological evaluation at the Carlo Besta Neurological Institute in Milan between January and October 2024. Neurophysiological testing included video-EEG polymyographic recordings (P-VEEG) and somatosensory evoked potentials (SEPs). Informed consent to perform video recordings was obtained in all patients. For each patient, anamnestic, clinical, and instrumental data were collected. The initial clinical categorization of each movement disorder (i.e., myoclonus, tremor, chorea, dystonia, tics, and dyskinesias) was assessed by experienced pediatric neurologists specialized in movement disorders at our tertiary referral center (GZ, FZ, FG, RS) before neurophysiological studies. This clinical classification was subsequently compared with that derived from neurophysiological findings. All patients were evaluated according to a standardized protocol including simultaneous video-EEG and polygraphic recording of MDs. EEG recordings were obtained using 21 scalp electrodes arranged according to the International 10–20 system (Fp1/Fp2, F7/F8, F3/F4, C3/C4, T3/T4, T5/T6, P3/P4, O1/O2, Cz, Pz, Fz, A1, and A2), combined with a single-camera video recording system. EMG activity was recorded from at least two pairs of antagonist muscles using bipolar surface electrodes. The electrodes were placed at least 3 cm apart, aligned along the muscle fiber direction. The choice of the muscles to explore was customized for each patient to record the most involved body segments and/or those showing hyperkinesias requiring characterization. Recordings were divided into two parts: the first aimed at assessing the EEG during wakefulness, both at rest and during standardized activation procedures (hyperventilation, intermittent photic stimulation), as well as observing possible hyperkinesias at rest; the second part included evaluation of motor activity during specific tasks such as posture maintenance (e.g., Mingazzini I, wrist extension, “wing beating”), isometric contraction, voluntary motor activation and execution of specific tasks appropriate to age of the patient (e.g., goal-directed movements, playing with cubes, writing, drawing, pouring water etc.). Distraction maneuvers and weight loading were applied when appropriate. Each test was performed for a minimum duration of 60 s to obtain the most accurate possible qualitative and quantitative measurements. For each P-VEEG recording, we evaluated organization of the background EEG activity, the presence of epileptiform abnormalities, and video recording of MD. EMG interpretation took into account multiple parameters, including presentation of the disorder (i.e., at rest, during posture maintenance, specific tasks, or movement); localization of manifestations (upper or lower limbs, trunk, cranial muscles), synchronicity over antagonist muscles, burst duration and frequency (in rhythmic or nearly rhythmic MDs), pattern of muscular recruitment. The neurophysiological assessment was performed in accordance with established principles of movement clinical neurophysiology, which have been shown to improve diagnostic accuracy and therapeutic decision-making in patients with hyperkinetic movement disorders (3, 5, 17, 18, 19, 20–22, 30, 32). EMG pattern classification was performed by three experienced clinical neurophysiologists (DC, BR and LC) with expertise in EEG and EMG interpretation. Recordings were reviewed jointly and the final classification was reached by consensus. Back-averaging analysis was performed in cases with EMG evidence of a myoclonic pattern. In addition, SEPs were performed in patients with clinical suspicion of jerky movements or neurophysiological evidence of myoclonic components in order to assess markers of cortical hyperexcitability (4). Furthermore, we decided to perform SEPs also in patients with tremor for comparison in age-matched patients. Patients were classified as SEP-related hyperexcitability cases when the peak-to-peak amplitude of early cortical response (N20p–P25p) exceeded 7.44 μV in at least one hemisphere following upper-limb stimulation. This threshold corresponded to the mean plus three standard deviations of our internal normative SEP dataset obtained from contralateral median nerve stimulation (normative N20p–P25p amplitude: 4.26 ± 1.06 μV) (23). Differences in distribution between clinical categorical variables among the EMG patterns were tested using Chi-square tests or Fisher’s exact test. Effect sizes were reported using Cramer’s *V*. Continuous variables (age at observation and age at onset) were analyzed using the Kruskal–Wallis test. When the Kruskal–Wallis test was significant, *post-hoc* pairwise comparisons were performed using the Mann–Whitney *U* test. Significant statistical differences were assessed with *p* < 0.05. Adjustment for multiple comparisons was performed using the Bonferroni method to control the family-wise error rate.

## Results

**Figure 1:**
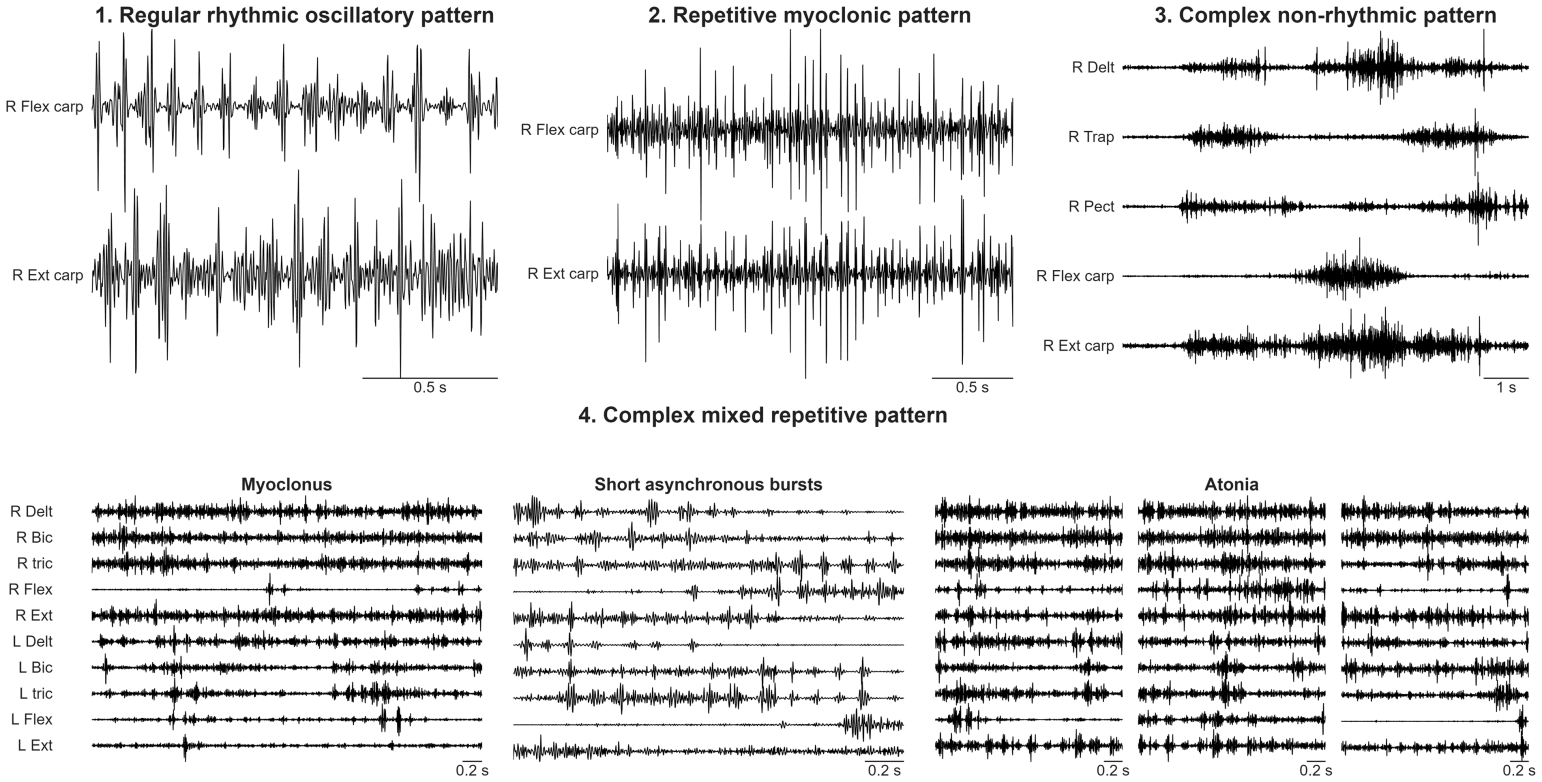
The figure illustrates the main EMG features of the four patterns analyzed in this paper: **(a)** Regular rhythmic oscillatory pattern (RRO) is characterized by rhythmic bursts involving synchronously or asynchronously antagonist muscles. **(b)** Repetitive myoclonic pattern (RM) is characterized by irregularly rhythmic brief EMG bursts usually at >12 Hz with consistently synchronous activation of antagonist muscles. **(c)** Complex non-rhythmic pattern (CNR) is characterized by irregular and highly variable EMG bursts involving synchronously or asynchronously antagonist muscles, often including tonic or phasic activity and possibly sustained co-contraction. **(d)** Complex repetitive pattern (CR) is characterized by the combination of repetitive oscillatory and irregular positive and negative components giving rise to a repetitive “myoclonic-tremor” at clinical inspection. Note the very short lapses of EMG activity resulting in brief intermittent atonias marked by asterisks. All repetitive patterns are present during voluntary activation of upper limbs (Mingazzini position). Case a: 14-year-old boy with essential tremor; Case b: 17-year-old girl with suspected progressive myoclonic epilepsy; Case c: 10-year-old boy with dystonia associated with PKAN mutation; Case d: 6-year-old boy with opsoclonus myoclonus.

**Figure 2:**
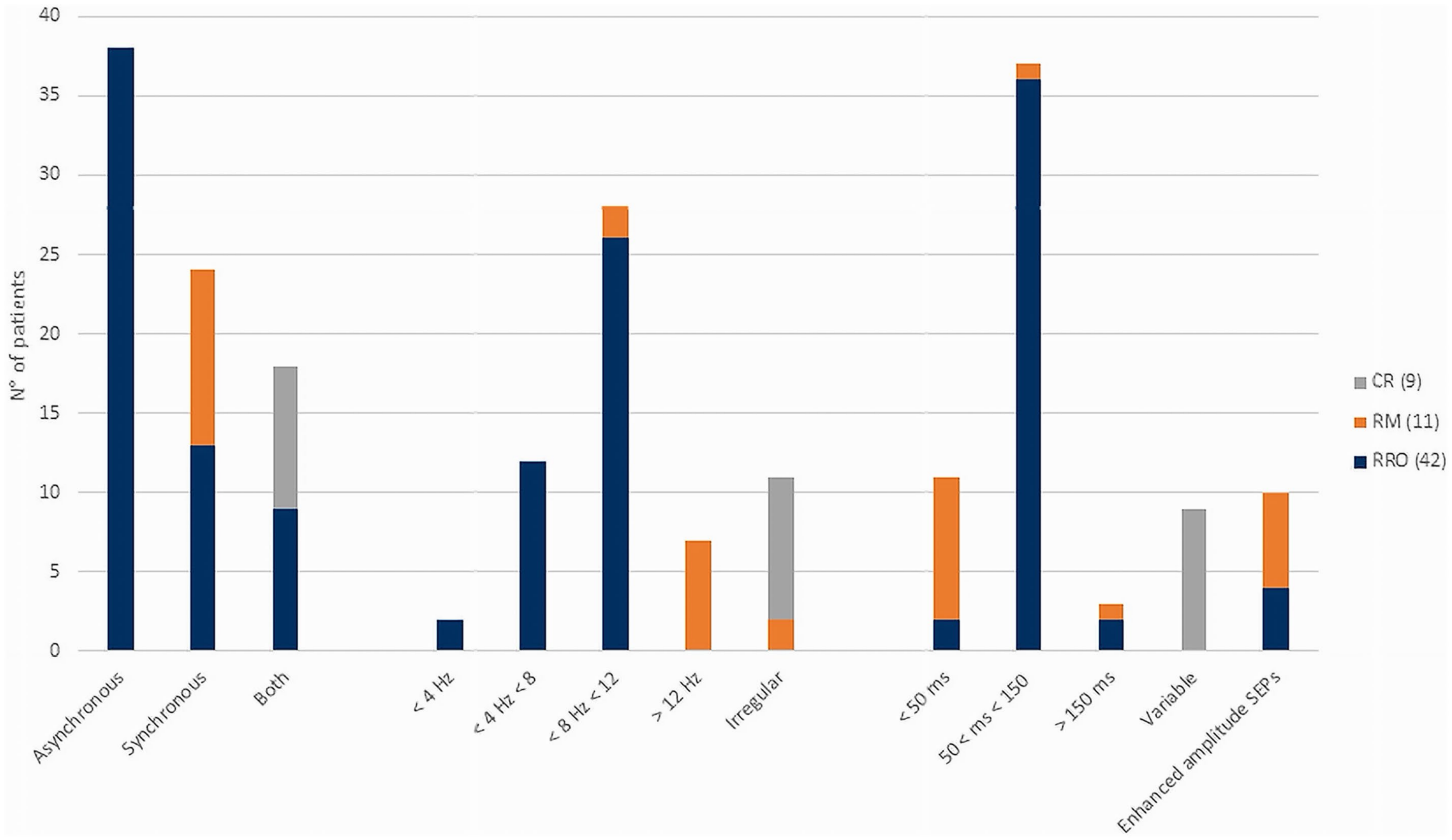
The figure illustrates the main features of repetitive EMG patterns: the regular rhythmic oscillatory pattern (RRO), the repetitive myoclonic pattern (RM), and the complex mixed repetitive pattern (CR). Significant differences were observed across patterns in terms of rhythmicity, frequency, burst duration, and the presence of signs of hyperexcitability. RM consistently showed synchronous activation, high frequency, and short burst duration. RRO and CR could present with either synchronous or asynchronous activation; however, CR was characterized by a more irregular pattern with variable burst duration. The number of patients included in each pattern group is reported in brackets.

**Figure 3:**
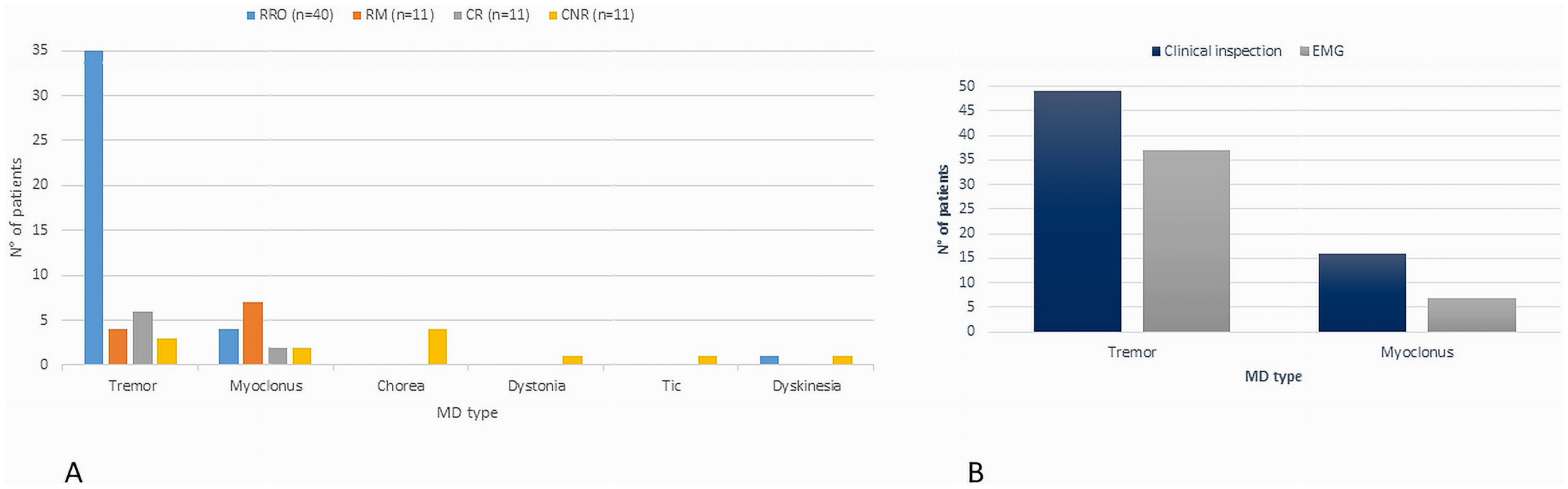
The figure shows movement disorders (MD) as assessed by clinical inspection and by P-VEEG analysis. Although a significant association between clinical diagnosis and EMG pattern was found (Fisher’s exact test, *p* = 0.003, Cramer’s *V* = 0.42), the distribution of EMG patterns across clinical syndromes was heterogeneous, suggesting a discrepancy between clinical inspection and EMG analysis **(A)**. This discrepancy is particularly pronounced in clinically defined tremor and myoclonus **(B)**.

**Figure 4:**
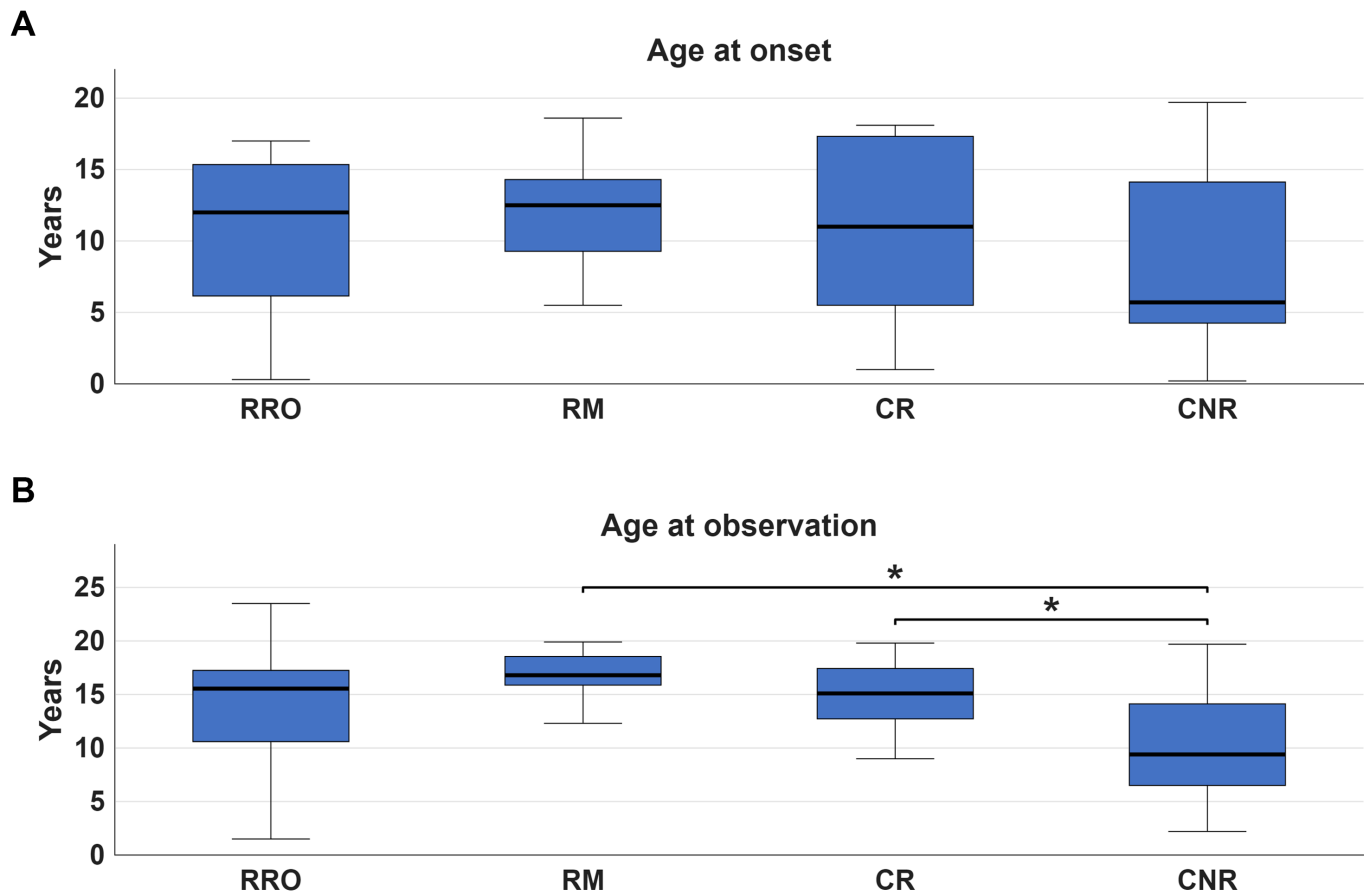
The figure shows the distribution of age across EMG patterns. No significant differences were found when comparing age at onset across EMG patterns (H(3) = 1.53, *p* = 0.675) **(A)**. In contrast, when comparing age at observation across EMG patterns, a significant effect was observed (H(3) = 9.12, *p* = 0.028) **(B)**, with significant differences between RM and CNR (*U* = 18.00, *p* = 0.004) and between CR and CNR (*U* = 22.00, *p* = 0.038). *p < 0.05.

Mean age at MD onset was 9.4 ± 6.1 years, and mean age at video-EEG polygraphy was 13.5 ± 5.1 years (mean delta onset: 4.1 ± 3.5 years). Age at recording was <6 years in 9 (12.3%), 6–14 years in 23 (31.5%), and >14 years in 41 (56.2%). A family history of movement disorders was present in 21.9% of cases, epilepsy in 5.5%, and other neuropsychiatric conditions in 26.0%. In 15 patients (20.5%) MD was identified during neurological evaluation performed for other reasons, thus the exact onset could not be determined. Clinical indication for neurophysiological test was tremor in 50 (68.5%), myoclonus in 15 (20.5%), chorea in 4 (5.5%), polymorphic dyskinesias in 2(2.7%), dystonia and tics in 1 case, respectively. In 23 patients (31.5%), a mild to severe interference with daily living activity was reported. Intellectual disability (ID) was present in 18 patients (24.7%) and developmental delay (DD) in 14 (19.2%). Epilepsy occurred in 18 patients (26.0%), including focal epilepsy in 5, generalized epilepsy in 12, combined focal and generalized in 2. Background EEG activity was normal in 58 (79.5%) patients; epileptiform abnormalities were detected in 16 (22.5%) patients. SEPs were performed in 51 patients showing signs of cortical hyperexcitability in 11 (21.6%) and other alterations such as increased cortical response latency in 9 (17.6%). Diagnostic tests disclosed the presence of genetic etiology in 13 (18%) subjects harbouring pathogenic variants in one of the following genes: *PRRT2, SCN1A, CHRNA7, NARP, HTT, NEU1, DYNC1H1*, Xq deletion, *NAA15, SEMA6B, ATM, PANK2, SCN8A*. Two cases were confirmed as having functional tremor and were not included in EMG analysis. Polygraphic analysis identified four distinct EMG patterns (Figure 1): (1) *regular rhythmic oscillatory pattern (RRO)*, consistent with tremor, in 40 patients (56.3%); (2) *repetitive myoclonic pattern* (RM) characterized by discrete, shock-like, EMG bursts, in 11 patients (15.5%); (3) *complex mixed repetitive pattern (CR)*, combining rhythmic oscillations, myoclonic bursts, and brief atonic phenomena, in 9 patients (12.7%); (4) *complex non-rhythmic pattern (CNR)* encompassing chorea, dystonia, tics, and other dyskinetic movements, was identified in 11 patients (15.5%). This pattern was characterized by highly variable EMG activity, combining tonic, phasic or tremulous bursts, possibly associated with synchronous or asynchronous muscle activation and/or sustained co-contraction. Significant differences were observed among the three repetitive/rhythmic EMG patterns (RRO, RM and CR, Figure 2) in agonist–antagonist synchrony (Fisher’s exact test, *p* < 0.001, Cramer’s *V* = 0.78), rhythmicity (Fisher’s exact test, *p* < 0.001, Cramer’s *V* = 0.81), burst duration (Fisher’s exact test, *p* < 0.001, Cramer’s *V* = 0.77), and the presence of SEP hyperexcitability (Fisher’s exact test, *p* = 0.002, Cramer’s *V* = 0.50). In 10 patients, the RM pattern showed features consistent with cortical myoclonus, including synchronous activation, high-frequency or irregular brief bursts, with both SEP with incresead amplitude and positive back-averaging in 6 patients. By contrast, the CR pattern showed variable synchrony and burst duration without SEP abnormalities. A strong significant association between clinical diagnosis and EMG pattern was found (Fisher’s exact test, *p* < 0.001, Cramer’s *V* = 0.49, Table 1), however, the concordance between clinical and neurophysiological definition varied (Figure 3). The strongest association was observed in the clinically defined tremor where 72.9% (35 out of 48) of patients demonstrated RRO pattern whereas a small fraction of patients (13 out of 48) presented with RM (*n* = 4) or CR (*n* = 6) or CNR (*n* = 3) patterns. In the clinically defined myoclonus subgroup, the findings were more heterogeneous: 46.7% (7 out of 15) exhibited a RM pattern, 4 exhibited RRO, 2 CR and 2 CNR patterns. A notably high specificity was observed for chorea, where all 4 clinical cases (100%) were associated with CNR polygraphic pattern. Other hyperkinetic movements, such as tics and dyskinesia, primarily aligned with non-rhythmic or complex rhythmic patterns, while a single case of dyskinesia recorded in this cohort presented with RRO (see Table 2 for the detailed features of patients with discordant findings). The comparison of the four different patterns (Table 1) showed significant association between RM and signs of cortical hyperexcitability at SEPs (Fisher’s exact test, *p* = 0.023, Cramer’s *V* = 0.38), but it did not survive Bonferroni correction. We found no correlations between EMG pattern and epilepsy presence/type, or EEG. Age at observation differed significantly across EMG patterns (H(3) = 9.12, *p* = 0.028, Figure 4). *Post-hoc* pairwise comparisons indicated that the CNR pattern was more prevalent in younger patients compared with RM (*U* = 18.00, *p* = 0.004) and CR (*U* = 22.00, *p* = 0.038) patterns. No significant differences were observed between EMG patterns with respect to age at onset of MDs.

**Table 1:** Comparison of the four EMG patterns showed significant associations with clinical diagnosis and the presence of cortical hyperexcitability signs on SEPs.

		**EMG patterns**					
		Total (*n* = 71)	RRO (*n* = 40)	RM (*n* = 11)	CR (*n* = 11)	CNR (*n* = 11)	Statistic
Clinical diagnosis	Tremor	48	35	4	6	3	Fisher’s exact test, *p* < 0.001, Cramer’s *V* = 0.49
	Myoclonus	15	4	7	2	2	
	Chorea	4	0	0	0	4	
	Dystonia	1	0	0	0	1	
	Tic	1	0	0	0	1	
	Dyskinesia	2	1	0	0	1	
Age at recording	<6 years	8	5	0	0	3	Fisher’s exact test, *p* = 0.189
	6-14 ears	22	12	2	3	5	
	>14 years	41	23	9	6	3	
Neurological examination	Normal	39	26	6	5	2	Fisher’s exact test, *p* = 0.049, Cramer’s *V* = 0.33
	Pathological	32	14	5	4	9	
Epilepsy	No	53	32	5	7	9	Fisher’s exact test, *p* = 0.123
	Yes	18	8	6	2	2	
Epilepsy type	Focal	5	3	0	1	1	Fisher’s exact test, *p* = 0.598
	Generalized	12	5	5	1	1	
	Combined	1	0	1	0	0	
Anti-seizures medication	No	49	32	4	5	8	Fisher’s exact test, *p* = 0.038, Cramer’s *V* = 0.35
	Yes	22	8	7	4	3	
Anti-spastic/dystonic medication	No	68	40	10	9	9	Fisher’s exact test, *p* = 0.114
	Yes	3	0	1	0	2	
EEG background activity	Normal	57	36	6	8	7	Fisher’s exact test, *p* = 0.112
	Altered	9	3	3	0	3	
	Destructured	5	1	2	1	1	
EEG abnormalities	No	55	33	6	7	9	Fisher’s exact test, *p* = 0.272
	Yes	16	7	5	2	2	
SSEP	Normal	31	21	3	4	3	Fisher’s exact test, *p* = 0.023, Cramer’s *V* = 0.38
	Hyperexcitability signs	11	4	6	0	1	
	Other alteration	9	4	1	3	1	

A weaker level of significance was observed when considering the use of antiseizure medications and anti-spastic/anti-dystonic treatments. No correlations were found between EMG pattern and the presence or type of epilepsy, or with EEG findings. No significant associations were found with the other variables analyzed. Two patients with functional tremor were not included in the statistical analysis.

**Table 2:** Detail of the features of the 22 cases with discordant findings between clinical evaluation and EMG findings.

	Pt number	Clinical syndrome	Age at onset of MD (y)	Age at recording (y)	Clinical indication	Polygraphic pattern	SSEP	Genetic etiology
Discordant tremors	1	Tremor spectrum	*U*	3.8	Tremor	CNR	n.p.	
	2	Focal epilepsy	11	14.9	Tremor	CR	n	
	3	Tremor spectrum	9	16.7	Tremor	RM	n	
	4	DEE	11.6	15.6	Tremor	RM	n	CHRNA7
	5	Generalized epilepsy	*U*	5.7	Tremor	CNR	n	
	6	Generalized Epilepsy	16.5	17.1	Tremor	CR	n.p.	
	7	Tremor spectrum	6	10.1	Tremor	CR	n	
	8	Generalized epilepsy	13	17.1	Tremor	RM	Increased amplitude	
	9	Generalized epilepsy	*U*	18.1	Tremor	CR	n	NEU1
	10	Tremor spectrum	1	13.6	Tremor	CR	n	
	11	Paroxysmal dyskinesia	4	8.9	Tremor	CNR	n	
	12	Generalized epilepsy	5.5	19.9	Tremor	RM	Increased amplitude	SCN8A
	13	Tremor spectrum	4	9.0	Myoclonus	CR	n	
Discordant myoclonus	14	Tremor spectrum	*U*	13.4	Myoclonus	RRO	n.p.	
	15	Opsoclonus myoclonus	4.8	4.9	Myoclonus	RRO	n	
	16	Tremor spectrum	*U*	15.6	Myoclonus	RRO	n.p.	
	17	DE	15.7	17.2	Myoclonus	RRO	n	NARP
	18	Ataxia Telangectasia	8	15.1	Myoclonus	CR	n.p	ATM
	19	Corea	*U*	12.0	Myoclonus	CNR	Increased amplitude	
	20	Dystonia	5	9.0	Myoclonus	CNR	n	PANK2
	21	Generalized epilepsy	*U*	17.2	Myoclonus	CR	n	
Dyscordant dyskinesia	22	Paroxysmal dyskinesia	*U*	11.0	Dyskinesias	RRO	n.p	PRRT2

DE, developmental encefapholopathy; DEE, developmental and epileptic encephalopathy.

## Discussion

In this study we applied a standardized neurophysiological protocol, combining video-EEG and surface EMG polygraphy, an easily applicable and highly precise tool to characterize hyperkinetic movement disorders in a large pediatric cohort. This approach has been historically applied mainly to paroxysmal MDs (15) and to the differential diagnosis with epilepsy (3). Our findings demonstrate that this method provides valuable objective markers that complement clinical evaluation, allowing more accurate classification of childhood movement disorders and improving diagnostic precision also in very young but cooperative children. Consistent with previous reports on polymyographic studies in hyperkinetic movement disorders (6), clinical inspection alone proved insufficient in a substantial proportion of cases. Diagnostic inconsistencies were particularly evident in patients initially classified as tremor or myoclonus, the most frequent hyperkinetic phenotypes in our cohort. Conversely, concordance was generally higher for less repetitive or non-rhythmic hyperkinetic disorders such as chorea, dystonia, tics, and polymorphic dyskinesias. Nevertheless, a small number of patients within this group showed EMG patterns consistent with tremor or myoclonus, indicating the usefulness of complementary neurophysiological studies even in conditions with clinically distinctive phenotypes (5). Diagnostic challenges in classifying MDs on clinical grounds alone have been recently highlighted in adult literature (5, 13, 20, 24). These studies have underscored how difficult it may be to distinguish irregular tremor, myoclonus, and mixed hyperkinesias, especially when movements are subtle, variable, or age-dependent. In our cohort, polygraphy identified either discordant patterns (e.g., tremor reclassified as myoclonus and vice versa) or complex rhythmic or non-rhythmic patterns characterized by the coexistence of tremor-like oscillations, myoclonic bursts, and brief atonic components, which were not detected by clinical inspection alone. To address this heterogeneity, we adopted a pragmatic polygraphic classification based on surface EMG features, grouping motor phenomena according to rhythmicity, regularity, and pattern complexity. This approach stemmed from the significant discordance observed between the clinical diagnosis and the EMG patterns, particularly in repetitive MDs. Consequently, this grouping is intended to complement clinical classifications by providing an objective, neurophysiology-based framework capable of capturing features that may be under-recognized during clinical inspection. Our findings revealed a “grey zone” in pediatric movement disorders, represented by the complex repetitive (CR) pattern, in which clinically apparent rhythmic or jerky movements may exhibit overlapping EMG features. In this context, the novelty of our study lies not in redefining the canonical EMG patterns, but in their systematic application to a pediatric cohort and their integration with clinical practice. In our cohort, polygraphic analysis resulted in a diagnostic shift in approximately 30% of cases. In most patients, polygraphy served a confirmatory role, supporting the initial clinical hypothesis. In discordant cases, neurophysiological characterization allowed refinement of the diagnostic work-up according to the updated classification. Conversely, the identification of complex repetitive pattern defined a subgroup of patients for whom both clinical and neurophysiological follow-up should be maintained, to allow reassessment and potential age-related reclassification. Overall, neurophysiology contributed primarily to diagnostic clarification and counselling rather than to immediate therapeutic changes. The need for an integrated approach is particularly evident in pediatric populations, where motor phenomenology is influenced by developmental factors, cooperation, and physiological variability. In this context, mixed or transitional patterns-such as those observed in the CR pattern- may reflect immature or dynamically interacting motor circuits rather than discrete disease entities (16). In our cohort older patients more frequently exhibited myoclonic or complex repetitive EMG patterns, whereas younger children more often showed non-rhythmic patterns. The interpretation of these age-related differences should be made with caution, as our cohort reflects a one-year recruitment period and is therefore subject to selection bias. Nevertheless, a consistent tendency toward a more fully expressed myoclonic phenotype in older patients was observed. Finally, the distinct EMG and SEP profiles observed across repetitive patterns further support their physiological heterogeneity. Myoclonic patterns showed the typical features of cortical myoclonus, including synchronous antagonist activation, brief high-frequency bursts, and association with increased SEP amplitude, consistent with cortical hyperexcitability of the sensorimotor cortex. EEG–EMG back-averaging was also applied and supported the presence of cortical correlates preceding the EMG bursts in 6/11 cases, further reinforcing the interpretation of these patterns as cortical myoclonus. This association is well recognized in adult cohorts (3, 4, 24, 25), but remains less explored in children. Conversely, the CR pattern exhibited variable synchrony and irregular rhythmicity without SEP abnormalities, suggesting the involvement of different or distributed motor generators rather than a purely cortical mechanism. In our cohort, 18 out of 73 patients (25%) had epilepsy. Pathogenic variants affecting genes related to channelopathies, synaptopathies, and metabolic disorders were identified in 13 patients, 7 of whom presented with both epilepsy and MDs. The association between epilepsy and MDs is well established, and several studies have demonstrated a shared genetic etiology (26, 27, 33). Within this framework, neurophysiology may play a role beyond differential diagnosis by providing an objective tool for refined phenotyping and for linking clinical manifestations to underlying pathophysiological mechanisms in genetically heterogeneous disorders. Accurate classification of movement disorders, particularly the distinction between myoclonus and tremor, is clinically crucial in patients with suspected progressive diseases, which often present in childhood or adolescence (28, 29). In this context, combined EEG–EMG recordings are essential, as the identification of myoclonic EMG patterns together with assessment of EEG background activity may enable early recognition of such disorders. Overall, our results underline the close interdependence between neurophysiological and clinical features and emphasize the importance of an integrated diagnostic approach. P-VEEG assessment should be routinely incorporated into the evaluation of ambiguous or complex pediatric hyperkinesias, as it substantially refines and standardizes clinical interpretation. At the same time, the limitations of neurophysiological approaches must be acknowledged, particularly in conditions with borderline features between tremor and rhythmic myoclonus. The intrinsic variability observed in children, evidenced by composite EMG patterns, highlights the need for pediatric-specific reference values and age-adjusted interpretative frameworks. This study has several limitations, including its retrospective design, heterogeneous etiologies, and variable ages at assessment, which may influence EMG expression, as well as the absence of formal inter-rater agreement analysis due to the consensus-based classification approach. Moreover, the assessments were conducted in the context of routine clinical neurophysiology and were therefore not formally blinded to the referral diagnosis. Finally, the generalizability of our findings, is limited as the distribution of the phenotypes in our cohort is not representative of the full spectrum of pediatric hyperkinetic MDs. This reflects the referral patterns to a tertiary center specialized in pediatric movement disorders, where neurophysiological studies are more frequently required for challenging cases with tremulous or jerky movement disorders. Future prospective studies with larger cohorts, homogeneous age ranges, and quantitative EMG measures will be essential to validate this classification and formalize developmental norms. Despite these limitations, this work represents one of the largest pediatric cohorts assessed with combined video-EEG and polygraphy and provides strong evidence for the systematic integration of neurophysiological tools into the diagnostic evaluation of childhood hyperkinetic movement disorders. The identification of reproducible EMG patterns opens the way for standardized electrophysiological classification schemes and more individualized, physiology-based clinical management.

## Data Availability

The raw data supporting the conclusions of this article will be made available by the authors, without undue reservation.
